# Supplementary material for: Novel endosomolytic compounds enable highly potent delivery of antisense oligonucleotides
Source: Commun Biol. 2022 Mar 1;5:185. doi: 10.1038/s42003-022-03132-2 (PMC8888659; doi:10.1038/s42003-022-03132-2)
Supplement: Supplementary file 2 — Supplementary Material [file 42003_2022_3132_MOESM2_ESM.pdf]

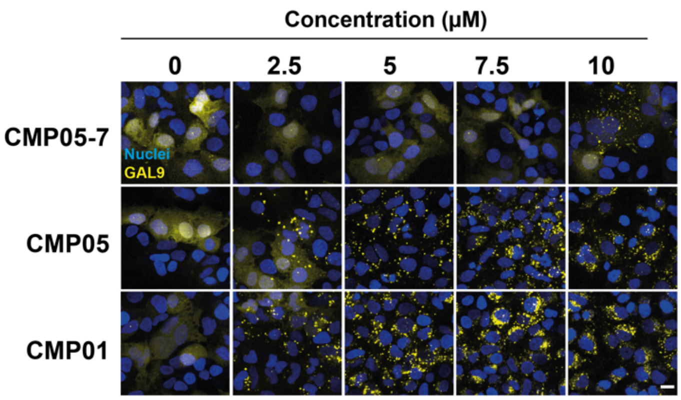

Supplementary Figure 1. Screening endosomolytic compounds for Gal9 body-inducing endosomal rupture. *HuH7_mCherry-Gal9 cells were treated with compound across 10-point dose titration series for 2 hr and then stained for nuclei (Hoechst 33342), fixed, and imaged. Representative images display the translocation of Gal9 from cytosolic to punctate in a dose-dependent manner. Scale bar = 20 μm.*

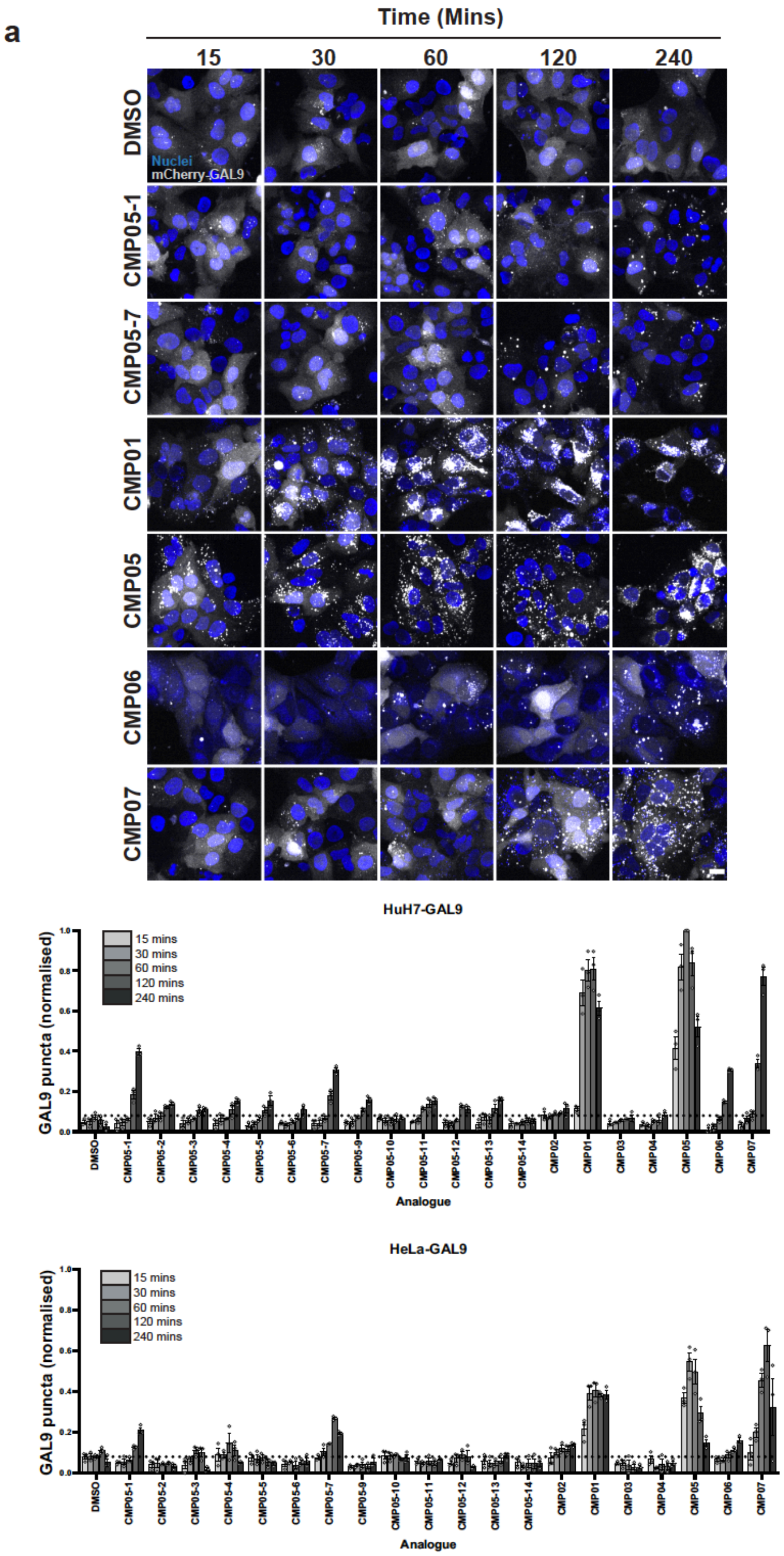

Supplementary Figure 2. Compound screening via Gal9-recruitment assay. ***a***. *HuH7_mCherry-Gal9 stable cells were treated for up to 4 hr with 5 µM of each compound. Images were acquired for up to*

4 hr of treatment duration. Scale bar = 20 µm. ***b,c***. *Quantitation of the Gal9 recruitment in HuH7 (b) and HeLa (c) reveals time-dependent endosomal rupture, even from compounds which did not exhibit detectable functional activity. Values are reported as puncta per relative nuclear signal and plotted as mean +/- SEM normalized to the highest overall induced signal (CMP05, 60min, HuH7) from n=3 separate experiments.*

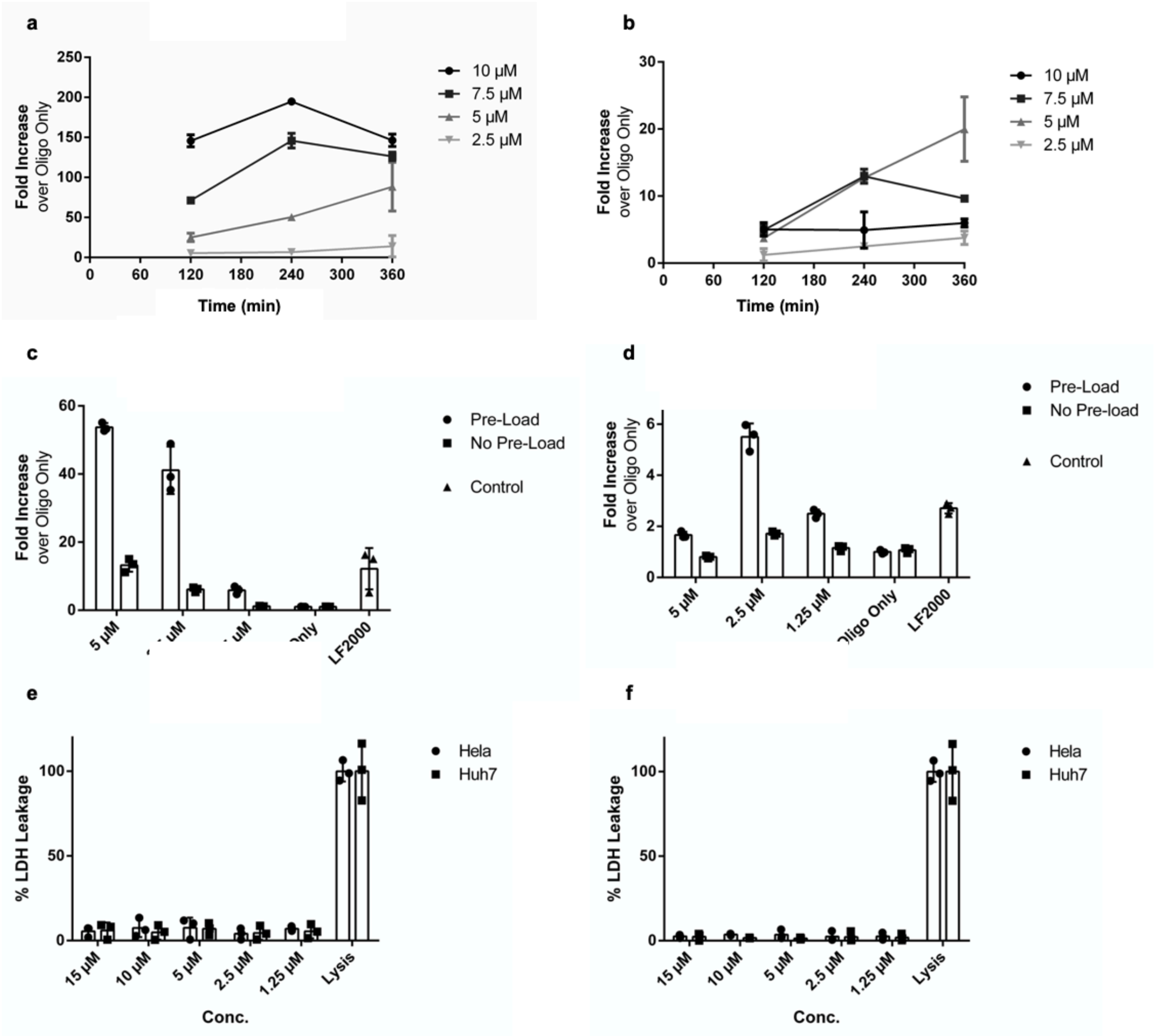

Supplementary Figure 3. Alternate treatment variables influence the efficacy of CMP05 and 705-SSO. *a,b. HeLa (a) and HuH7 (b) cells were treated with 1 μM SSO similarly to the treatment plan described in figure 3, with the exception that the pre-loading step was performed for 48 hr instead of 24. Cells were analyzed after 2, 4, and 6 hr of co-treatment with CMP05. Values are reported as fold increase over naked oligo-only treatment. Data comes from n=3 experiments. c,d. HeLa (c) and HuH7 (d) cells in the "Pre-Load" group were treated similarly to the treatment plan described in figure 3b, while cells in the "No Pre-Load" group were only co-treated with CMP05 and 1 μM SSO simultaneously for 2 hr. Values are reported as fold increase over naked oligo-only treatment. Lipofectamine 2000 was used as a positive transfection control. e,f. Media lactate dehydrogenase (LDH) levels taken after 2 hr of compound treatment reveal no significant acute toxicity from CMP05 (e) or CMP05-7 (f) in either HeLa or HuH7 wildtype cells. Values are reported as percentage of LDH release upon treatment with lysis buffer as a positive control.*

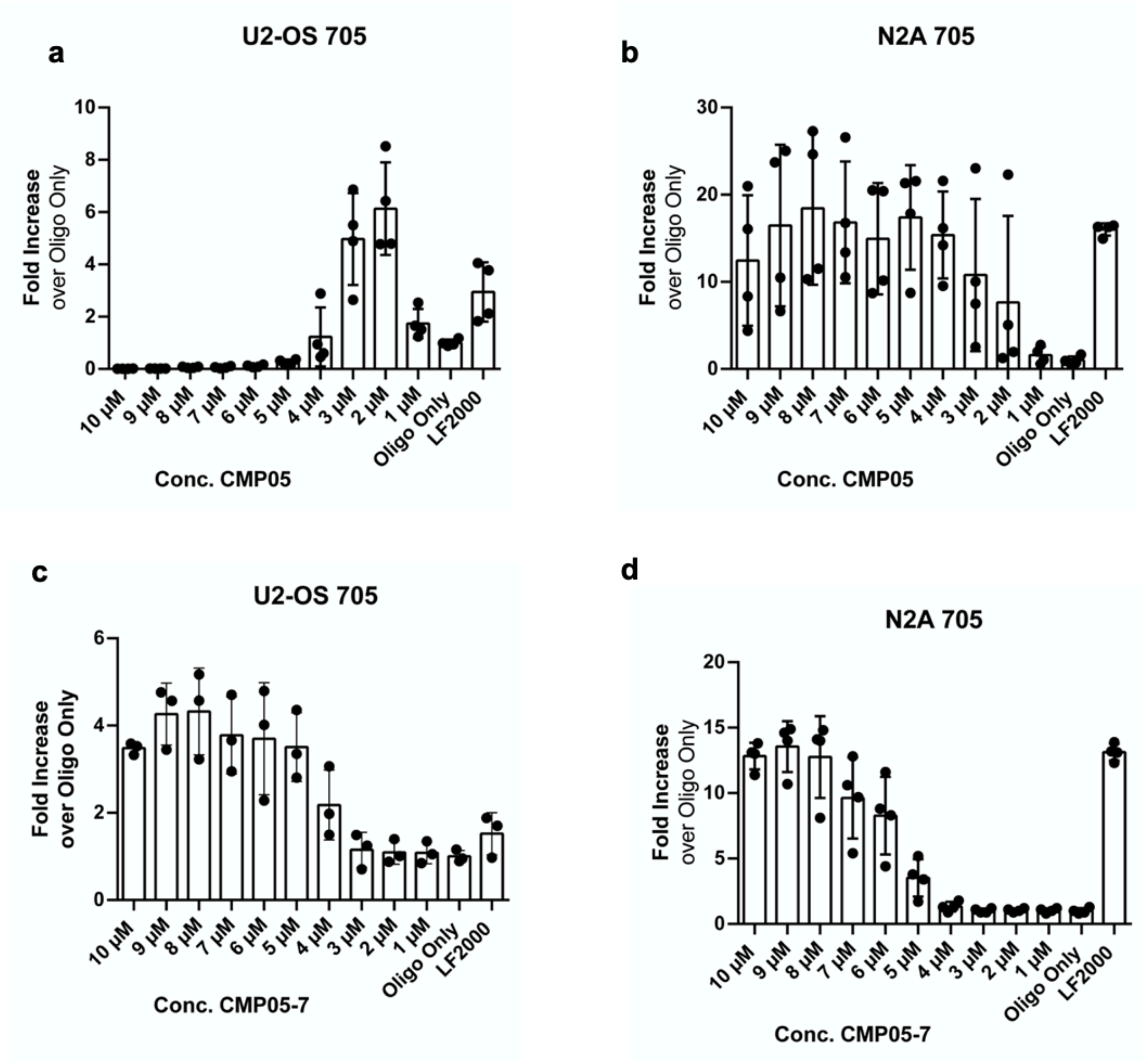

Supplementary Figure 4. Compound-induced SSO activity is observed across multiple cell lines. ***a,b.*** *U2-OS (a) and N2A (b) cells were treated with 1 µM SSO and varying concentrations of CMP05 and analyzed for activity. Values are reported as fold increase over oligo-only treatment. Data comes from n=3 experiments.* ***c,d****. U2-OS (c) and N2A (d) cells were treated with 1 µM SSO and varying concentrations of CMP05-7 and analyzed for activity. Values are reported as fold increase over oligo-only treatment. Data comes from n=3 experiments.*

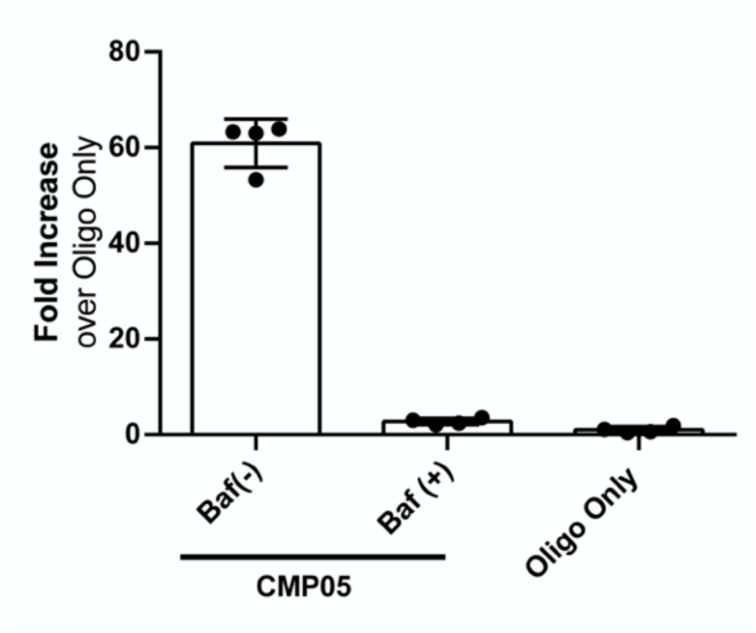

Supplementary Figure 5. Bafilomycin inhibits compound-induced SSO activity. *HeLa cells were treated with 1 µM SSO similarly to the treatment plan described in figure 3, with the exception that 2 µM bafilomycin was co-treated with 5 µM CMP05 in the Baf (+) condition. Values are reported as fold increase over naked oligo-only treatment. Data comes from n=3 experiments.*

a

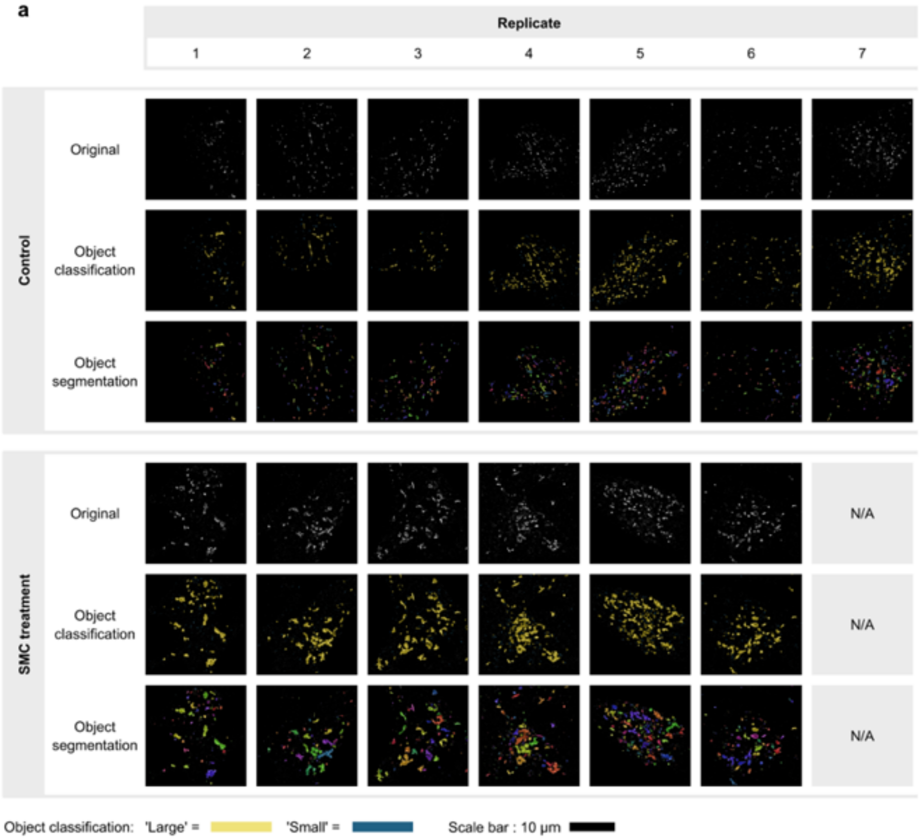
Replicate
1
2
3
4
5
6
7
Control
Original
Object classification
Object segmentation
SMC treatment
N/A
Object classification: 'Large' = 'Small' = Scale bar : 10 μm

b

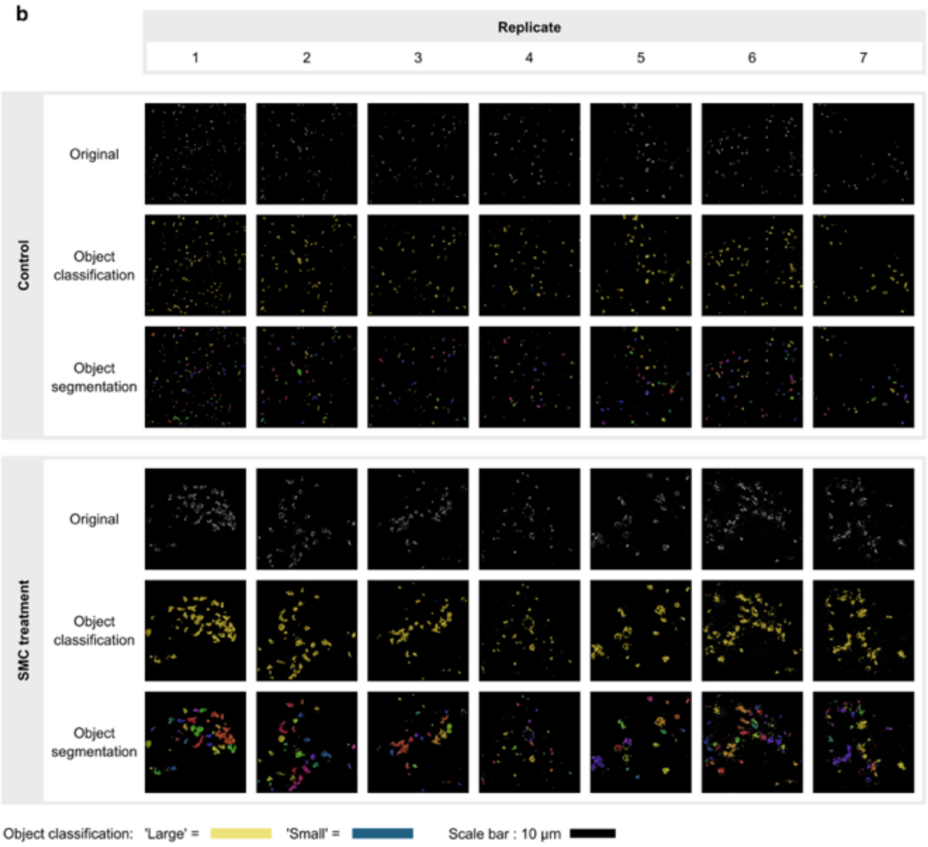
Replicate
1
2
3
4
5
6
7
Control
Original
Object classification
Object segmentation
SMC treatment
Object classification: 'Large' = 'Small' = Scale bar : 10 μm

Supplementary Figure 6. STORM Endosomal characterization and quantitation with ilastik image analysis software. *a, b. STORM images from HeLa (a) and HuH7 (b) were used to define an ilastik pipeline which identifies LAMP1-positive endosomes. This pipeline generates quality control images of the object-level segmentation and makes multiple measurements of the shape of the binary objects. The validity of the segmentation was assessed qualitatively by inspecting the quality control images.*

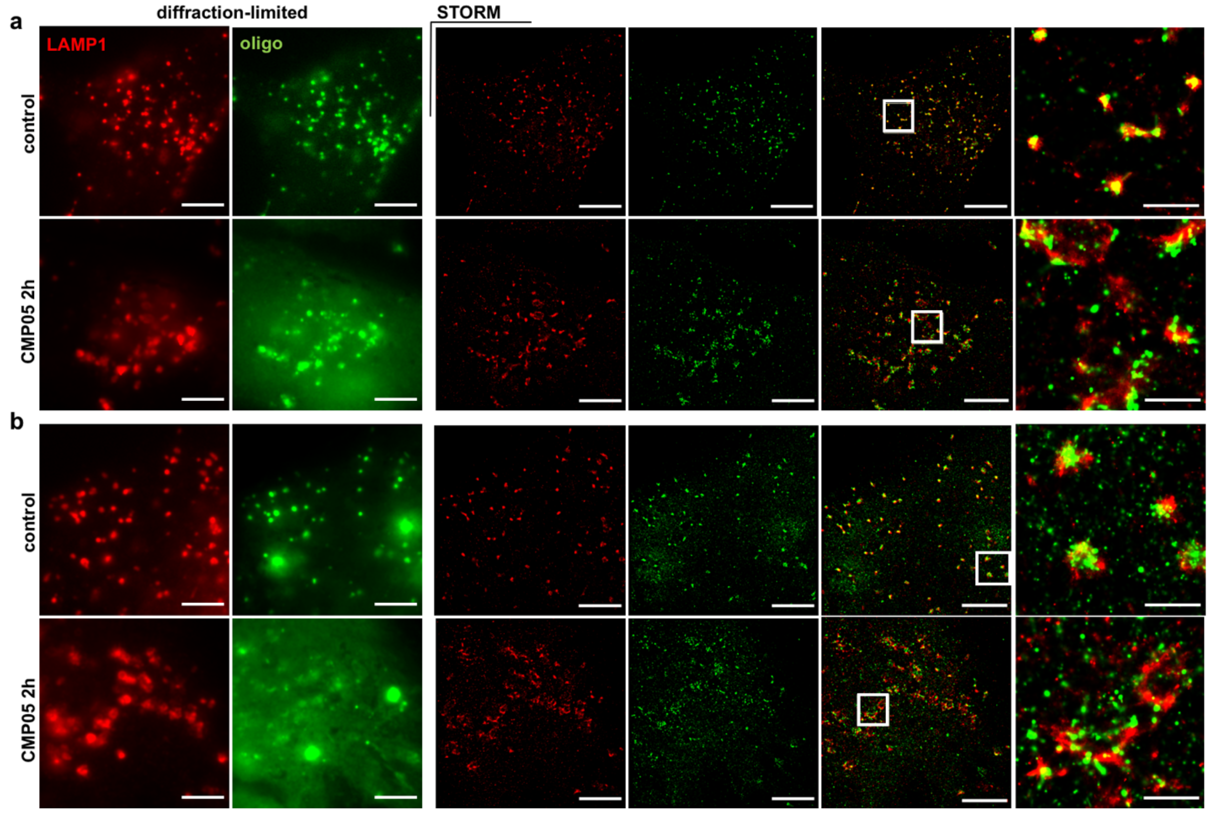

Supplementary Figure 7. Super-resolution imaging of SSO trafficking in CMP05 treated HeLa and HuH7 cells. *a. Diffraction-limited and STORM images of lysosomes (red; LAMP1 ICC) and Alexa Fluor 568 labelled oligonucleotide (green) in control HeLa cells and after 2 hr treatment with 5 µM CMP05. Scale bars 5 µm, except in the zoomed area 1 µm. b. Diffraction-limited and STORM images of lysosomes (red; LAMP1 ICC) and Alexa Fluor 568 labelled SSO (green) in control HuH7 cells and after 2 hr treatment with 2.5 µM CMP05. Scale bars are 5 µm, except in the zoomed area 1 µm.*

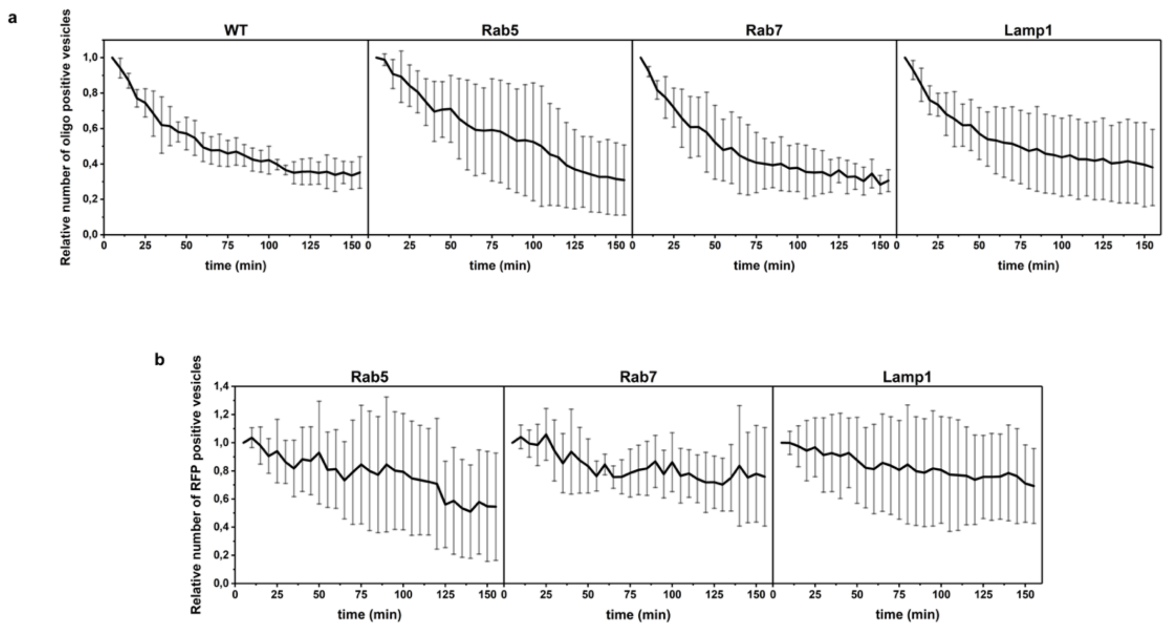

Supplementary Figure 8. Quantitation of live-cell confocal SSO-endosome colocalization assay. *a,b. Relative number of SSO-positive (a) and (m)RFP-positive (b) vesicles in HuH7 cells (wt or cells overexpressing mRFP-Rab5, mRFP-Rab7 or Lamp1-RFP) after a 20 min pulse-incubation with 1 µM SSO and 2.5 µM CMP05. The cells were imaged every 5 min during continuous treatment with 2.5 µM CMP05 and particles were detected using the ComDet plugin to ImageJ (see figure 6 and accompanying text). The results are presented as number of particles normalized to the first image in each set and to the DMSO-treated control sample ±SD. The experiment was repeated on six separate occasions (N=6). Samples containing cells displaying CMP05-originating toxicity within the field of view were removed from the analysis and analysis was hence performed on N=3-6 (CMP05: WT n=3, Rab5 n=4, Rab7 n=4, Lamp1 n=5; DMSO: WT, Rab5, Rab7 and Lamp1 n=6).*

Supplementary Methods

Chemical Synthesis of CMP05 and analogues

General procedure for preparation of intermediate **2**

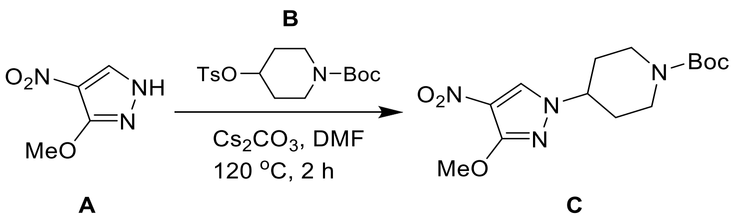

Reactants **A** (9.00 g, 62.8 mmol, 1.00 *eq*) and **B** (28.8 g, 81.1 mmol, 1.29 *eq*) were added to a flask charged with DMF (63 mL). Cs_2CO_3 (30.5 g, 93.7 mmol, 1.49 *eq*) was added to the mixture, which was then degassed with nitrogen and stirred at 120 °C for 2 h. TLC showed full conversion of reactant **A**. The pH of the reaction mixture was adjusted to approx. 7-8 by adding HCl (1 M) in portions followed by extraction into DCM (3 times). The combined DCM layer was washed with brine twice before it was dried over $MgSO_4$. DCM was evaporated *in vacuo* after filtering off $MgSO_4$. The crude residue was purified by column chromatography (SiO_2, Petroleum ether/Ethyl acetate=30/1 to 3/1) leaving intermediate **C** (18.1 g, 70.5% yield, 80.0% purity) as a yellow solid.

^{1}H NMR (400 MHz DMSO-d_6): δ 8.746 (s, 1H), 7.94 (s, 1H), 4.69-4.68 (m, 1H), 4.31-4.26 (m, 2H), 4.05 (s, 3H), 3.33 (s, 4H), 2.88 (s, 3H), 2.72 (s, 1H), 2.01-1.98 (m, 2H), 1.81-1.74 (m, 2H), 1.40 (s, 9H)

General procedure for preparation of intermediate **D**

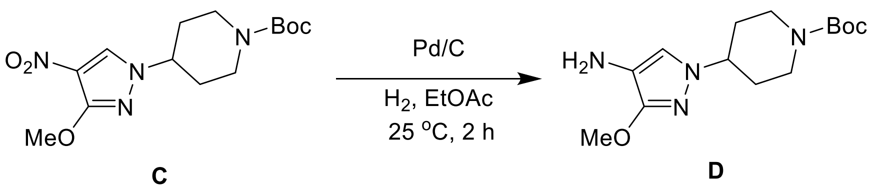

Intermediate **C** (17.0 g, 52.0 mmol, 1.00 *eq*) was dissolved into EtOAc (110 mL) then Pd/C (1.70 g, 52.0 mmol, 10% purity, 1.00 *eq*) was added. The reaction mixture was hydrogenated at 1 bar and ambient temperature for 10 h. TLC showed full conversion of intermediate **C**. The catalyst was removed by filtration through Celite then EtOAc was evaporated *in vacuo*. The crude residue was purified by column chromatography (SiO_2, Petroleum ether/Ethyl acetate=30/1 to 3/1) leaving intermediate **D** (10.9 g, 70.6% yield) as black blue oil.

^{1}H NMR (400 MHz DMSO-*d*$_6$): δ 7.00 (s, 1H), 4.01-3.94 (m, 3H), 3.52 (s, 3H), 2.88-2.73 (m, 2H), 1.88-1.84 (m, 2H), 1.66-1.60 (m, 2H), 1.40 (s, 9H)

General procedure for preparation of intermediate **G**

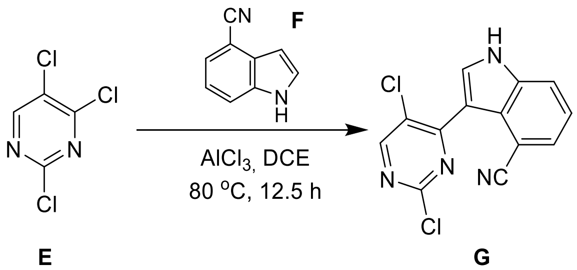

Reactant **E** (4.84 g, 26.3 mmol, 1.50 *eq*) was dissolved into DCE (100 mL) then $AlCl_3$ (3.52 g, 26.3 mmol, 1.44 mL, 1.50 *eq*) was added to the mixture. The mixture was stirred at 80 °C for 0.5 hr then reactant **F** (2.5 g, 17.59 mmol, 1.00 *eq*) was added to the mixture, causing the

initial yellow colour to change to deep orange. The mixture was stirred at 80 °C for 12 h. LC/MS confirmed formation of intermediate **G**. The mixture was quenched by addition of ice-water (200 mL) then **G** was extracted into EtOAc (250 mL x 3). The EtOAc layer was washed with brine twice before the solvents were removed *in vacuo*. The residue was purified by column chromatography (SiO_2, Petroleum ether/Ethyl acetate=30/1 to 1/1). Intermediate **G** (0.50 g, 9.83% yield) was isolated as an orange solid.

^{1}**H NMR** (400 MHz DMSO-d_6): δ 8.95 (s, 1H), 8.37 (s, 1H), 7.91 (d, J = 8.0 Hz, 1H), 7.69 (d, J = 8.0 Hz, 1H), 7.41 (t, J = 8 Hz, 1H).

General procedure for preparation of intermediate **H**

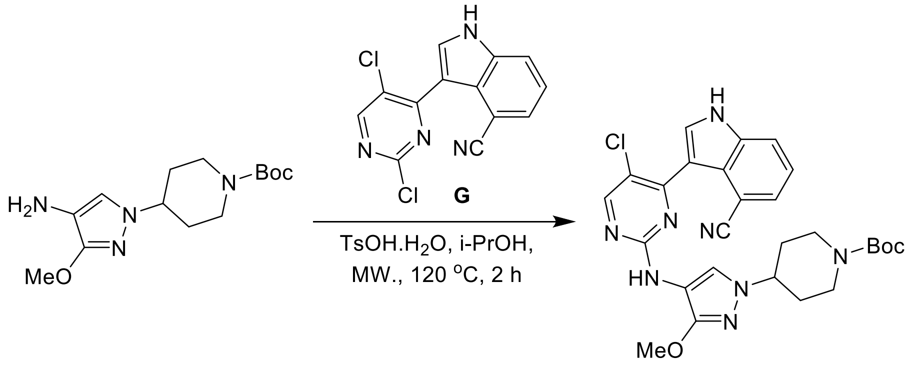

D **H**

Intermediates **D** (0.50 g, 1.69 mmol, 1.00 *eq*) and **G** (444 mg, 1.54 mmol, 0.91 *eq*) were dissolved into *i*-PrOH (10.0 mL) in a microwave vial. TsOH (529 mg, 3.08 mmol, 1.82 *eq*) was added to the mixture then the reaction mixture was heated in a microwave reactor at 120 °C for 2 h. LC/MS shows intermediate **H** formed. The pH of the mixture was adjusted to 7-8 with sat. $NaHCO_3$. Cmpd 4-1 was then into EtOAc (50 mL x 3), washed with brine twice before EtOAc was evaporated leaving intermediate **H** (0.40 g, crude) as a yellow solid.

General procedure for preparation of Compound **CMP05**

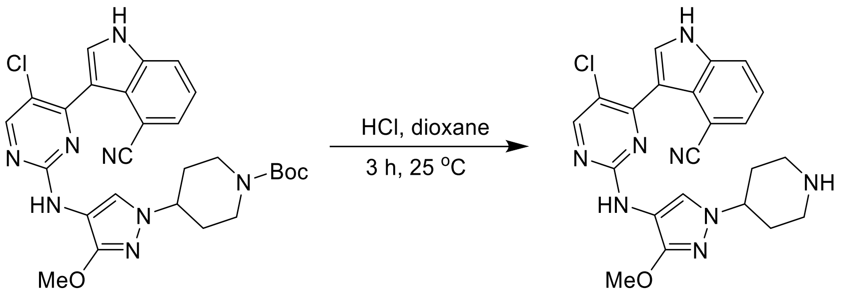

H **CMP05**

Intermediate **H** (0.90 g, 1.64 mmol, 1.00 *eq*) was dissolved into HCl/dioxane (4 M, 9.00 mL, 21.9 *eq*) and left stirring 3 hr at 25 °C. LC/MS showed formation of **CMP05**. Sat. $NaHCO_3$ (50.0 mL) was added carefully to the solution until pH = 8. The product was extracted into EtOAc (100.0 mL x 2), then the organic layer was dried over Na_2SO_4, filtered and concentrated *in vacuo*. Purification by prep-HPLC (column: Phenomenex Gemini-NX 80*40mm*3um;mobile phase: [water(10mM NH4HCO_3)-ACN];B%: 20%-40%,8min) yielded **CMP05** (0.062 g, 8.34% yield) as white solid.

Analysis data for Compound CMP05

Approximated apparent pKa values:

1. pKa (HL/H+L) = 12.76 +/- 0.30
2. pKa (H2L/H+HL) = 9.76 +/- 0.10
3. pKa (H3L/H+H2L) = 0.73 +/- 0.10
4. pKa (H4L/H+H3L) = -1.03 +/- 0.10

^{1}H NMR (400 MHz DMSO-d_6): δ 8.44(s, 1H), 8.08 (s, 1H), 7.85 (d, *J* = 8.0 Hz, 1H), 7.73 (s, 1H), 7.61 (d, *J* = 8.0 Hz, 1H), 7.34 (t, *J* = 8.0 Hz ,1H), 3.90 (s, 1H), 3.77 (m, 3H), 2.96 (s, 2H), 1.84 (s, 2H), 1.67-1.60 (m, 2H).

m/z (ES+), [M+H]+ = 449.1

Analysis data for Compound CMP05-7

Approximated apparent pKa values:

1. pKa (HL/H+L) = 14.59 +/- 0.30
2. pKa (H2L/H+HL) = 9.77 +/- 0.10
3. pKa (H3L/H+H2L) = 0.66 +/- 0.10
4. pKa (H4L/H+H3L) = -0.83 +/- 0.10

^{1}H NMR (400 MHz DMSO-d_6): d ppm 11.82 (br s, 1 H), 8.48 (s, 1 H), 8.30 (s, 2 H), 7.70 (s, 1 H), 7.46 (d, *J*=7.54 Hz, 1 H), 7.18 (t, *J*=8.01 Hz, 1 H), 6.91 - 7.10 (m, 1 H), 4.15 (q, *J*=7.10 Hz, 2

H), 3.97 (dd, *J*=15.64,7.54 Hz, 1 H), 3.02 (d, *J*=18.84 Hz, 2 H), 2.55 - 2.67 (m, 2 H), 1.84 - 2.00 (m, 2 H), 1.56 - 1.84 (m, 2 H), 1.22 (t, *J*=6.97 Hz, 3 H)

m/z (ES+), [M+H]+ = 438.0
