# Supplementary material for: Novel endosomolytic compounds enable highly potent delivery of antisense oligonucleotides
Source: Commun Biol. 2022 Mar 1;5:185. doi: 10.1038/s42003-022-03132-2 (PMC8888659; doi:10.1038/s42003-022-03132-2)
Supplement: Supplementary file 3 — Reporting Summary [file 42003_2022_3132_MOESM3_ESM.pdf]

nature portfolio

Corresponding author(s): Jeremy Bost
Last updated by author(s): Jan 10, 2022

Reporting Summary

Nature Portfolio wishes to improve the reproducibility of the work that we publish. This form provides structure for consistency and transparency in reporting. For further information on Nature Portfolio policies, see our Editorial Policies and the Editorial Policy Checklist.

Statistics

For all statistical analyses, confirm that the following items are present in the figure legend, table legend, main text, or Methods section.

n/a	Confirmed	
☐	☒	The exact sample size (n) for each experimental group/condition, given as a discrete number and unit of measurement
☐	☒	A statement on whether measurements were taken from distinct samples or whether the same sample was measured repeatedly
☐	☒	The statistical test(s) used AND whether they are one- or two-sided<br>*Only common tests should be described solely by name; describe more complex techniques in the Methods section.*
☐	☒	A description of all covariates tested
☐	☒	A description of any assumptions or corrections, such as tests of normality and adjustment for multiple comparisons
☐	☒	A full description of the statistical parameters including central tendency (e.g. means) or other basic estimates (e.g. regression coefficient) AND variation (e.g. standard deviation) or associated estimates of uncertainty (e.g. confidence intervals)
☐	☒	For null hypothesis testing, the test statistic (e.g. F, t, r) with confidence intervals, effect sizes, degrees of freedom and P value noted<br>*Give P values as exact values whenever suitable.*
☒	☐	For Bayesian analysis, information on the choice of priors and Markov chain Monte Carlo settings
☒	☐	For hierarchical and complex designs, identification of the appropriate level for tests and full reporting of outcomes
☐	☒	Estimates of effect sizes (e.g. Cohen's d, Pearson's r), indicating how they were calculated

Our web collection on statistics for biologists contains articles on many of the points above.

Software and code

Policy information about availability of computer code

Data collection	MetaMorph and Micro-manager were used during STORM acquisition.
Data analysis	STORM imgaes were reconstructed with the ThunderSTORM pugin for FIJI. STORM was then segmented using ilastik and objects segmented using CellProfiler. Data structuring and quantitation from STORM was performed with R. Gal9 assay images were processed using Columbus and data was processed in Spotfire. Images were assembled using FIJI. Endosomal colocalization assay images were analyzed with ImageJ. Functional data from luciferase assays was analyzed and presented using GraphPad Prism.

For manuscripts utilizing custom algorithms or software that are central to the research but not yet described in published literature, software must be made available to editors and reviewers. We strongly encourage code deposition in a community repository (e.g. GitHub). See the Nature Portfolio guidelines for submitting code & software for further information.

Data

Policy information about availability of data

All manuscripts must include a data availability statement. This statement should provide the following information, where applicable:

- Accession codes, unique identifiers, or web links for publicly available datasets
- A description of any restrictions on data availability
- For clinical datasets or third party data, please ensure that the statement adheres to our policy

The data that support the findings of this study (data DOI: 10.5281/zenodo.5833599) are openly available for download under Creative Commons Attribution 4.0 International license under the repository name "Novel endosomolytic compounds enable highly potent delivery of antisense oligonucleotides" at https://doi.org/10.5281/zenodo.5833599

Field-specific reporting

Please select the one below that is the best fit for your research. If you are not sure, read the appropriate sections before making your selection.

☒ Life sciences ☐ Behavioural & social sciences ☐ Ecological, evolutionary & environmental sciences

For a reference copy of the document with all sections, see nature.com/documents/nr-reporting-summary-flat.pdf

Life sciences study design

All studies must disclose on these points even when the disclosure is negative.

Sample size	All experiments were performed in cell culture with approximately 10,000 cells per 100 microliters. Every functional experiment was performed on at least 3 of these wells per experiment, and this further performed at 3 separate experiments. Imaging experiments were performed in similar cellular ranges, however with less experimental replications in some cases.
Data exclusions	No data was excluded from analysis.
Replication	No measures were needed to verify reproductibility.
Randomization	Randomization was not used.
Blinding	Blinding was not relevant as this was a study in cellular biology.

Reporting for specific materials, systems and methods

We require information from authors about some types of materials, experimental systems and methods used in many studies. Here, indicate whether each material, system or method listed is relevant to your study. If you are not sure if a list item applies to your research, read the appropriate section before selecting a response.

Materials & experimental systems

n/a	Involved in the study	
☐	☒	Antibodies
☐	☒	Eukaryotic cell lines
☒	☐	Palaeontology and archaeology
☒	☐	Animals and other organisms
☒	☐	Human research participants
☒	☐	Clinical data
☒	☐	Dual use research of concern

Methods

n/a	Involved in the study	
☒	☐	ChIP-seq
☒	☐	Flow cytometry
☒	☐	MRI-based neuroimaging

Antibodies

Antibodies used	LAMP1 (D2D11, Rabbit mAb, Cell Signaling Technology, cat no. 9091S); donkey anti-rabbit IgG Alexa Fluor 647 (Invitrogen, cat no. A-31573)
Validation	Antibodies were validated by the manufacturers and experimentally optimized for our use in the required experiments.

Eukaryotic cell lines

Policy information about cell lines

Cell line source(s)	HeLa, Huh7
Authentication	None of the cell lines used were authenticated
Mycoplasma contamination	All cell lines were routinely tested for mycoplasma
Commonly misidentified lines (See ICLAC register)	N/A
